# BRCA2 Promotes Spontaneous Homologous Recombination In Vivo

**DOI:** 10.3390/cancers13153663

**Published:** 2021-07-21

**Authors:** Adam D. Brown, Scott Greenman, Alison B. Claybon, Alexander J. R. Bishop

**Affiliations:** 1 Department of Cellular Systems and Anatomy, University of Texas Health San Antonio, San Antonio, TX 78229, USA; brownad2012@gmail.com; 2 Greehey Children’s Cancer Research Institute, University of Texas Health San Antonio, San Antonio, TX 78229, USA; alison.claybon@gmail.com; 3 Department of Exercise Sciences, Brigham Young University, Provo, UT 84602, USA; sgreenman1987@gmail.com; 4 Mays Cancer Center, University of Texas Health San Antonio, San Antonio, TX 78229, USA

**Keywords:** BRCA2, homologous recombination, pink-eyed unstable, mouse

## Abstract

**Simple Summary:**

Inherited mutation of either BRCA1 or BRCA2 is associated with familial breast and ovarian cancer. As a tumor suppressor, BRCA2 functions to maintain genome stability but we do not know the genomic impact of this deficiency on normal tissues in normal contexts, rather than cancer cells that carry other gene mutations. Numerous murine models of BRCA2 have been developed to investigate BRCA2 tumor suppressor function but have been met with difficulties due to the lethality of most of these models during embryogenesis. To circumvent embryonic lethality here, we employ the cre conditional system combined with an assay for measuring changes in stability of a large tandem DNA repeat by homology-directed recombination. The relevance of this assay is that it can measure DNA recombination events in a normal growing/developing tissue. Here, we show that BRCA2 is required for homologous recombination in a normal developing tissue long before cancer arises.

**Abstract:**

Background: BRCA2 is known to be a tumor suppressor involved in homologous recombination repair and presumed to prevent genome instability in normal tissues prior to the development of tumors. Typical assessment of BRCA2 deficiency on the genome involves cell-based models using cancer cells with mixed genetic contexts, but the role in normal tissue in vivo has not been clearly demonstrated. Methods: Using conditional deletion of *Brca2* exon 11, the region containing all eight BRC repeats, in the retinal pigment epithelium and the pink-eyed unstable mouse model, we evaluate the frequency of DNA deletion events. Results: In the current study, we show that conditional loss of *Brca2* exon 11 results in a decreased frequency of spontaneous homologous recombination compared to wild-type mice. Of note, we observe no apparent concomitant increase in events that indicate single-strand annealing by the pink-eyed unstable mouse model. Conclusions: Therefore, our results demonstrate that BRCA2, as expected, is required for high-fidelity homologous recombination DNA repair in normal tissues, here in a tissue undergoing normal proliferation through normal development.

## 1. Introduction

Accurate replication of DNA is essential for maintaining genome stability and the prevention of malignant transformation. During DNA synthesis, the replication machinery can encounter a multitude of lesions from both exogenous and endogenous sources that could lead to fork stalling, fork collapse and strand breaks. It is suggested that spontaneous DNA double-strand breaks (DSBs) can arise at stalled replication forks, and the repair of these lesions often relies on a homologous recombination (HR) event utilizing the sister chromatid as a template. Furthermore, cells lacking the ability to undergo HR accumulate DSBs following replication, resulting in cell death [1]. The necessity for HR, particularly at the organismal level, is exemplified by the embryonic lethality observed following the removal of the mammalian RecA homolog RAD51 that is responsible for homology pairing during HR [2].

Similar to the embryonic lethality of the RAD51 knockout mouse, initial studies deleting the tumor suppressor gene *Brca2* (breast cancer 2, early onset gene) in mice by a targeted deletion that encompasses the eight BRC repeats within exon 11 also resulted in early embryonic lethality, and cells cultured from these embryos exhibited poor proliferation with sensitivity to DNA damaging agents [3,4]. Subsequent mouse models in which portions of the BRC repeats were present could partially rescue the lethality, though primary embryonic fibroblasts derived from these mice still proliferated poorly in culture [5,6]. Additionally, these fibroblasts were found to have a defect in DNA repair with increased chromosomal abnormalities yet retained normal levels of apoptosis [5]. Both RAD51 and BRCA2 expression correlate with cellular proliferation [7], and RAD51 was found to interact with BRCA2 at two independent sites (the BRC repeats internal to the protein within exon 11 and the c-terminal region of BRCA2) [4,8]. BRCA2-compromised cells fail to form RAD51 nuclear foci following the induction of DNA damage [9]. Direct evidence that BRCA2 has a role in HR was demonstrated using tissue culture systems reporting that BRCA2 is involved in the repair of spontaneous and DSB-induced lesion within direct repeats in a variety of different BRCA2 mutant cell lines. The conclusions drawn from these studies were that BRCA2 promotes error-free HR (e.g., RAD51-dependent) while suppressing error-prone homology-directed repair (HDR) (e.g., single-strand annealing [SSA]) [10,11,12]. Taken together, these studies suggest that BRCA2 is involved in maintaining genome stability, particularly in response to damage [13], presumably through its involvement in HR.

While the in vitro (tissue culture) data are in agreement, the requirement of BRCA2 for mouse development impedes our ability to test the in vivo function. Our laboratory previously incorporated a conditional system for measuring spontaneous HDR events in vivo by combining the pink-eyed unstable (*p^un^*) mouse model with a tissue-specific *Cre/loxP* recombinase system to conditionally delete genes of interest [14]. This strategy allows the examination of the effect of essential genes on HDR in vivo. To summarize, the in vivo HDR assay used the *p^un^* mouse model results from a tandem duplication of approximately 70 kb within the murine pigmentation *p* gene (also referred to as *Oca2*). Following the removal of one of these repeats, a nonfunctional *p* gene is converted into a single copy functional wild-type allele that can be observed phenotypically as a pigmented spot on the fur or retinal pigment epithelium (RPE). Based on studies by our laboratory [14,15,16,17], as well as studies using an analogous model in yeast [18], it is most likely that the deletion/reversion of a large tandem repeat (2 × 70kb) such as in the *p^un^* allele is a HDR-mediated event. Furthermore, our results to date suggest that the *p^un^* model can detect both RAD51-dependent (e.g., HR events such as unequal gene conversion or crossover events that occur in S-phase to G2-M) and -independent (e.g., SSA) events; this is based on *Parp1* deletion and *Blm* deletion resulting in an increase in mainly multi-cell spots (clonal expansion indicating the event occurred in an actively dividing cell and thus likely replication-tied) while *Brca1* mutation leaves only single-cell events (indicating an event not necessarily associated with replication) [14,17].

The relevance for such a system to understand BRCA2 function is appreciated due to the association of germline inheritance of a single mutated *BRCA2* gene with the increased risk of developing certain cancers, particularly breast and ovarian. Furthermore, the FANCD1 sub-group of DNA repair-defective Fanconi Anemia patients is actually the result of bi-allelic *BRCA2* mutation [13]. Our in vivo model will allow for investigational studies to better understand the consequences of loss of BRCA2 on HR prior to transformation or in the absence of other cancer-associated mutations in order to allow further exploration of potential therapeutic modalities (e.g., synthetic lethality via PARP1 inhibition in HR-deficient cancers). Here, we report a decrease in spontaneous HR events resulting from the in vivo conditional loss of *Brca2* exon 11 confirming cell-based results that BRCA2 promotes HR. Furthermore, this decrease appears to be due to a reduction in the events that we have classified as being replication tied, and therefore RAD51 dependent. This result aligns with our study of PARP1 deletion increasing RAD51-dependent HR events, further adding to the evidence for how synthetic lethality works. Finally, we observed no concomitant increase in SSA events in contrast to what has been reported by others using cell-based systems [10,11,12].

## 2. Materials and Methods

### 2.1. Mouse Lines

C57BL/6J and C57BL/6J *p^un/un^* mice were obtained from the Jackson Laboratory (Bar Harbor, ME). Mice carrying the exon 11 floxed allele *Brca2^tm1Brn^* (hereafter referred to as *Brca2^flx11^*) [19] were obtained from the Mouse Models of Human Cancer Consortium (MMHCC) mouse repository (Frederick, MD, USA). Cre was expressed in the mouse RPE using the *Trp1-Cre* transgenic mouse line [20] obtained from Dr. P. Chambon, and Cre activity was observed using the nuclear localized beta-galactosidase reporter knock-in mouse line *RC::PFwe* [21] from Dr. S. Dymecki. Lastly, the early embryonic Cre-expressing mouse line *Ella-Cre* was obtained from Dr. P. Leder. All mice were made C57BL/6J *p^un/un^* congenic by first backcrossing five times with C57BL/6J and then two backcrosses with C57BL/6J *p^un/un^*. Conditional (*Brca2^flx11/flx11^*) and constitutive (*Brca2^wt/Δ11^*) colonies were established and maintained similar to the conditional and constitutive colonies described by Brown et al. [14]. Crossing of these two cohorts produced offspring that are either conditional heterozygous (*Brca2^wt/co11^*) or conditional null (*Brca2^Δ11/co11^*) for exon 11. All animal studies were carried out in accordance with University and Institute IACUC policies, as outlined in protocol 07005-34-02-A, B1, C.

### 2.2. Obtaining and Scoring Reversion Events of the Retinal Pigment Epithelium

Eyes were harvested and dissected to isolate wholemount RPEs according to methods previously described [17], and Cre activity was observed using methods described in [14]. Dissected RPE whole mounts were visualized, imaged and scored on a Zeiss Lumar version 12 stereomicroscope, Zeiss AxioVision MRm camera and Zeiss AxioVision 4.6 software (Thornwood, NY, USA). The occurrence of and the number of cells making up a revertant eye spot, as well as the color of the nucleus following beta-galactosidase staining (i.e., clear or blue) were recorded. Manual assessment of Cre activity was visualized via the percentage of RPE nuclei with blue stain as described in [14].

### 2.3. RNA Isolation and Reverse Transcription-PCR (RT-PCR)

RNA was isolated from wild-type and BRCA2 delta 11 heterozygote male testis using the RNeasy^®^ kit according to manufacture protocol (QIAGEN^®^ Germantown, MD, USA). To obtain cDNA, we used the ImProm-II™ Reverse Transcription System kit (Promega Fitchburg, WI, USA). In brief, 2 µg of total RNA was used in the first-strand cDNA synthesis reaction. The final concentration of each component of the 30 µL reverse transcription reaction was ImProm-II™ 5× reaction buffer [1.3×], MgCl2 [2.5 mM], dNTP mix [0.67 mM], RNasin^®^ ribonuclease inhibitor [20 U] and 1 µL of ImProm-II™ Reveserve Transcriptase. Ten microliters of the first-strand cDNA synthesis was added to the 30 µL RT reaction, and carried out using the thermal cycle of 25 °C for 5 min, 42 °C for 60 min and 70 °C for 15 min. For the PCR, we used the GoTaq^®^ Flexi DNA polymerase system (Promega Fitchburg, WI, USA). The final concentrations of each component of the 50 µL PCR was 5× GoTaq^®^ Flexi buffer [1×], MgCl2 [2.5 mM], dNTP [0.2 mM each], primer [1 µM each], GoTaq^®^ polymerase and 2 µL of cDNA template. The PCR thermal cycle was 1 cycle of 95 °C for 2 min; 30 cycles of 95 °C for 1 min, 60 °C for 1 min, 72 °C for 2 min; 1 cycle of 72 °C for 5 min. The primers used were either taken from Evers et al. [22] or designed. Primer sequences used for the 67 bp amplicon spanning exons 10 and 11 were (5′-GAAGCAAGTGCTTTTGAAG-3′ and 5′-CAGAAGAATCTGGTATACCTG-3′) and for the 362 bp amplicon spanning exons 10 and 14 were (5′-GAAGCAAGTGCTTTTGAAG-3′ and 5′-ACGTCGTGAGCCGGTAAGATTGAA-3′). Amplicons were resolved on a two percent agarose gel and visualized by UV following ethidium bromide staining.

### 2.4. Protein Isolation and Immunoprecipitation

Whole protein lysate was isolated from wild-type and BRCA2 delta 11 heterozygote mouse testis using SDS-free RIPA buffer and mechanical homogenization, and protein was quantified using the Bradford assay. Equal quantity (0.5 mg) of protein was used for immunoprecipitation with anti-BRCA2 (ab90541) that recognizes the carboxy-terminal (c-terminal) domain of BRCA2. Each sample was eluted off of protein-A beads with equal volumes of 2× loading buffer. Membranes were probed with anti-BRCA2 (sc21174) that also recognizes the c-terminal domain and detected using standard methods.

### 2.5. Statistical Analysis

All statistics were performed using GraphPad Prism (La Jolla, CA, USA). These include tests for normality (Shapiro–Wilk test), equal variances (*F*_max_ test), 2-group comparison (Mann–Whitney test), and multi-group comparison (Kruskal–Wallis test) with multiple comparisons (Dunn’s test) and a contingency test (Fisher exact test).

## 3. Results

### 3.1. Establishment of the Constitutive p^un^ Brca2 Cohort

To facilitate our conditional *p^un^* assay system, we previously utilized a strategy of crossing a constitutive and conditional cohort for our gene of interest [14]. As the literature on BRCA2 indicates that the BRC repeat containing exon 11 may be key to the protein’s interaction with RAD51 and its involvement in HR [23], we elected to use the same exon 11 deletion allele for both our constitutive and conditional cohorts. To establish a constitutive *Brca2* exon 11 deletion allele, we crossed *Brca2^wt/flx11^ p^un/un^* mice to *Ella-Cre^tg/tg^ p^un/un^*. The adenovirus *EIIa* promoter is active during the single-cell zygote stage of the preimplanted embryo [24] allowing for Cre excision of the *Brca2* conditional allele in the germline. Resultant *Brca2^wt/Δ11^ Ella-Cre^tg/o^ p^un/un^* offspring were then crossed to *p^un/un^* animals. All pups that inherited the *Brca2* delta 11 allele and not the *EIIa-Cre* allele were considered to have been the product of a germline deletion of *Brca2* exon 11 derived from the prior generation (data not shown). The *Brca2^wt/Δ11^* allele segregated at the expected Mendelian ratio (data not shown). These *Brca2^wt/Δ11^ p^un/un^* animals were then used to establish the constitutive cohort required for our conditional *p^un^* assay system with the final cohort comprised of animals that are *Brca2^wt/Δ11^ Trp1-Cre^tg/tg^ p^un/un^*, henceforth described as *Brca2^Δ11^* mice. In comparison, the conditional cohort that consists of *Brca2^flx11/flx11^ RC::PFwe^ki/ki^ p^un/un^* mice will be referred to as *Brca2^co11^* mice following excision by Trp1-Cre recombinase.

**Figure 1:**
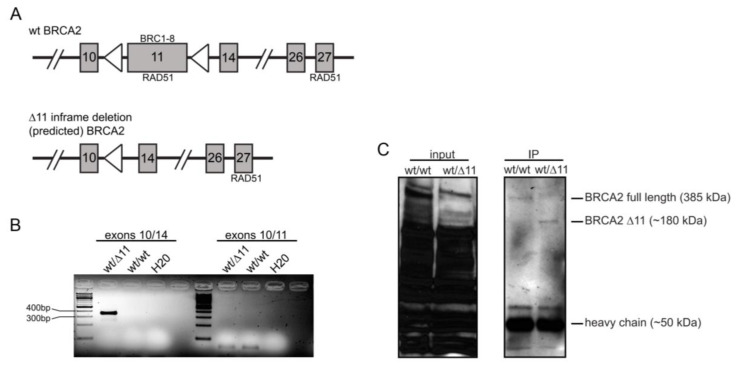
Brca2 delta 11 isoform. (**A**). Schematic of mouse wild-type (wt) *Brca2* and *Brca2* delta 11 deletion following Cre activity. Hatched lines represent excluded genomic regions and triangles (loxP sites). RAD51 interacts with BRCA2 at two distinct domains (i.e., the BRC repeats located in exon 11 and the c-terminal region of BRCA2) as noted. (**B**). PCR amplification of cDNA from BRCA2 wild-type and delta 11 isoform mouse testis for products encompassing exons 10 and 14 (362 bp product) and exons 10 and 11 (67 bp product). (**C**). Western blot depicting full-length BRCA2 and delta 11 deletion isoform protein products immunoprecipitated from mouse testis whole-cell lysates (original full Western Blot image provided in Figure S1).

The mRNA from the *Brca2* delta 11 allele has previously been reported to be expressed in a mouse mammary tumor model and is predicted to be in frame (Figure 1A) [22]. However, to date, there have been no reports successfully detecting a BRCA2 delta 11 protein product, likely due to the limited number of antibodies available to detect mouse BRCA2 protein. To determine whether we could observe the delta 11 isoform, we examined both mRNA and protein from *Brca2* delta 11 constitutive heterozygous and wild-type mouse testis. Using PCR primers either previously described or designed for this study, we were able to detect a PCR product encompassing exons 10 and 14 of *Brca2* using cDNA from *Brca2* delta 11 heterozygote and not wild-type samples (Figure 1B). The absence of a band between these two exons in wild-type samples would be expected due to the 3594 bp size of exon 11 [25]. As a control, we were able to detect a PCR product encompassing exons 10 and 11 from both samples (Figure 1B). Using immunoprecipitation prior to Western blot analysis, we were able to observe a band at approximately the correct molecular weight of the predicted BRCA2 delta 11 protein, as well as full-length BRCA2 protein, from testis of *Brca2* delta 11 heterozygous mice (Figure 1C), suggesting the production of a stable BRCA2 delta 11 protein.

### 3.2. Brca2 Delta 11 Conditional Heterozygosity Does Not Alter HR Frequency

**Figure 2:**
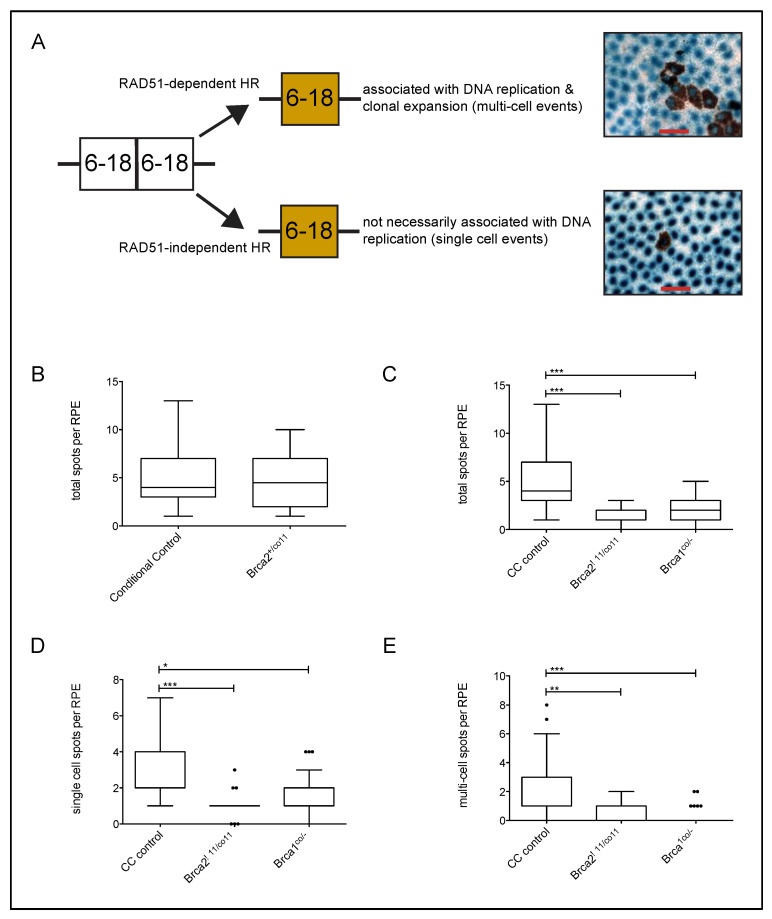
Full-length BRCA2 promotes high-fidelity homologous recombination. (**A**) Within the *p* gene results in an unpigmented eye spot (white box). Following a HR event, one of the duplicated regions is lost and pigmentation is visible (brown box). To the right are examples of a multi-cell (top) and a single-cell (bottom) reversion event (i.e., brown pigmented eye spots). Blue spots are nuclei and report Cre activity. Red bar indicates 50 µm. (**B**). To test for haploinsufficiency regarding HR, eye spots with blue nuclei from RPEs exhibiting ≥ 80% blue staining were analyzed for HR frequency. No significant difference (*p* = 0.82; Mann–Whitney test) was detected when *Brca2* delta 11 conditional heterozygosity was compared to controls. (**C**). The conditional loss of both alleles of *Brca2* exon 11 results in a significant decrease (*p* < 0.0001; Kruskal–Wallis test) of HR frequency compared to combined conditional (CC) controls and is similar to the conditional loss of *Brca1* (see Table 1 and text for details). Only those eye spots with blue nuclei from RPEs exhibiting ≥ 80% blue stain were used for analysis, and data are represented as a Tukey box and whisker plots (*** *p* < 0.001). The frequency of single-cell (**D**) and multi-cell (**E**) reversion events were plotted for eye spots with blue nuclei from RPEs exhibiting ≥ 80% blue staining from CC control and BRCA2 and BRCA1 experimental animals. Both single- and multi-cell events in the BRCA2 experimental group were significantly decreased compared to control (*p* < 0.0001; Kruskal–Wallis test). Data are represented as a Tukey box and whisker plots with the middle line representing the median, the box representing the inter-quartile range (i.e., 25th and 75th percentiles) and the whiskers the largest and smallest values not determined to be outliers from all data points within each comparison (* *p* < 0.05, ** *p* < 0.01, *** *p* < 0.001).

*BRCA2* heterozygosity is associated with a cancer susceptibility phenotype. The tumors derived from such carriers exhibit loss of heterozygosity, resulting in the loss of the remaining wild-type allele [26], suggesting that a single functional allele of *BRCA2* is sufficient to suppress any tumor development. Supporting this are the observations that mice heterozygous for *Brca2* mutations appear normal and do not form cancer [3,4,5]. To test whether or not *Brca2* delta 11 heterozygosity affects HR frequency in vivo, we compared spontaneous HR frequency between our conditional control (*RC::PFwe^co/wt^*) and conditional heterozygote (*Brca2^wt/co11^*) samples. The average frequency of each group was approximately five eye spots per RPE (Table 1), and therefore no difference was detected (*p* = 0.82; Mann–Whitney test) (Figure 2B). Furthermore, the spontaneous HR frequency for *Brca2* delta 11 conditional heterozygotes in this study is similar to that of earlier studies from our laboratory including *Blm* (the genes associated with Bloom’s syndrome) and *Brca1* conditional heterozygote samples [14]. This result suggests that our *Brca2* delta 11 conditional heterozygotes are not deficient in the repair of spontaneous damage, at least not via HR.

### 3.3. Conditional Loss of Full-Length BRCA2 Results in Decreased HR Frequency

Based on the in vitro evidence for a role of BRCA2 in HR and the embryonic lethality observed in BRCA2 null mice, we applied a tissue specific conditional deletion system that we developed to measure the frequency of HR in vivo for essential genes. In our previous study using this conditional system, we found that the nuclear localized beta-galactosidase reporter for Cre activity was also a reliable indicator for excision of our gene of interest and that increased Cre activity in the experimental animals correlated very well with altered HR phenotype; the most striking HR phenotype was observed when we restricted our analyses to RPEs with greater than 80% beta-galactosidase activity (i.e., blue stain) [14]. Similar to that early study, the RPEs examined here displayed differing degrees of beta-galactosidase staining (data not shown). Using our most stringent criteria, we only report those results from RPEs with the greatest Cre activity (RPEs with >80% beta-galactosidase staining). To test for any difference, we compared the frequency of eye spots per RPE in our conditional *Brca2* delta 11 null samples to control (Table 1). Samples from the conditional control and conditional heterozygotes were combined for this analysis because no difference was found between them (Figure 2A). The average frequency of the combined control was approximately five eye spots per RPE compared to less than two eye spots per RPE for the conditional *Brca2* delta 11 null samples (Table 1) and found to be significantly different (*p* < 0.001; Dunn’s Multiple Comparison test) (Figure 2C). It should be noted that similar results were also found when HR frequencies were calculated from reversion events regardless of beta-galactosidase staining (i.e., eye spots with and without a blue nuclei) and only those reversion events positive for beta-galactosidase (i.e., eye spots with blue nuclei) (data not shown). *BRCA1* is another breast cancer susceptibility gene that has been shown to promote HR [12,14,27,28], as well as interact with BRCA2 in replicating cells [29]. Therefore, we next compared the frequency of HR events between our conditional deleted *Brca2* delta 11 and *Brca1* null samples and found that HR frequencies were equally decreased in both (Figure 2C). Taken together, our results are in agreement with earlier in vitro studies stating that BRCA2 promotes HR, though here we have found that the BRC repeats within BRCA2 are necessary for this function.

Reversion events can be classified as either being single- or multi-cellular (defined as an eye spot comprised of >2 RPE cells) (Figure 2A). Our laboratory has reported that the loss of Poly (ADP-ribose) polymerase (PARP1) or BLM leads to an overall increase in HR frequency and that absence of either of the proteins results in a proportional increase in multi-cell reversions compared to wild type [14,17]. Furthermore, BRCA1 conditional null RPEs had a decreased HR frequency with a proportional decrease in multi-cell reversions compared to wild type [14]. These observations, in combination with the knowledge of mouse eye development [30], lead us to hypothesize that multi-cell reversions are associated with replication and classified as being RAD51 associated (e.g., gene conversion) (Figure 2A). In contrast, it would seem that single-cell reversions are not dependent upon replication, are not necessarily associated with cellular proliferation or clonal expansion and are most likely the result of a RAD51-independent event (e.g., SSA) (Figure 2A). Therefore, we measured the proportional difference in single- and multi-cell reversions in our *Brca2* delta 11 conditional null samples and compared them to our controls and *Brca1* conditional null samples. The trend for *Brca2* delta 11 conditional null samples was similar to the *Brca1* conditional null samples compared to controls in that the multi-cell events were proportional decreased. We further went on to quantify the frequency of single- and multi-cell events per RPE. As expected, a significant difference was detected for both single- (*p* < 0.001; Dunn’s Multiple Comparison Test) and multi-cellular (*p* < 0.01; Dunn’s Multiple Comparison Test) events in *Brca2* delta 11 conditional null samples compared to control and no difference between BRCA2 and BRCA1 samples (Figure 2D,E). Although we observe a decrease in both classes of reversion events (single- and multi-cell), it is interesting to note that the decrease in our *Brca2* delta 11 conditional null samples for multi-cell events was more significant than the decrease in single-cell events, the converse of what we observed with conditional deletion of *Brca1*.

## 4. Discussion

Tumor cells that are defective in either of the breast cancer susceptibility genes (i.e., *BRCA1* or *BRCA2*) are sensitive to the inhibition of PARP1 [31,32]. More recently, this inhibition was also shown to have antitumor activity with limited adverse side effects in cancer patients with *BRCA1* or *BRCA2* mutations [33]. This synthetic lethal approach assumes the following tenets: (1) replicating cells defective in either BRCA1 or BRCA2 have decreased capacity in the repair of DNA by HR; (2) replicating cells that retain one functional allele of either BRCA1 or BRCA2 retain their ability to repair DNA damage by HR; and (3) replicating cells defective in PARP1 or inhibited for its activity have increased levels of DNA damage that can be repaired by HR. Studies in our laboratory have set out to provide in vivo animal model data to support this therapeutic approach. To date, we have shown that the constitutive loss of PARP1 or conditional loss of BRCA1 results in the increase and decrease in HR, respectively, and these HR events are likely to be RAD51-dependent [14,17]. Additionally, HR was not affected in mice that were heterozygous for either PARP1 or BRCA1 [14,17]. Herein, these studies are extended to test whether the conditional loss of BRCA2 also fits this model.

We first used our conditional system to assess the affect of *Brca2* delta 11 heterozygosity on HR. We found that the conditional loss of exon 11 in one allele of *Brca2* did not affect spontaneous levels of HR compared to control (Figure 2B). This finding is in agreement with mouse models showing that heterozygous mice do not differ from wild type [3,5,6], and that the basal levels of DNA damage and RAD51 focus formation in BRCA2 heterozygote cell lines do not differ from controls [34]. These results suggest that the retention of one functional allele of BRCA2 is sufficient for cells to cope with spontaneous damage, at least those that are normally repaired by HR.

We next found that BRCA2 loss of function, through conditional excision of the BRC repeats located in exon 11, resulted in a significant reduction in the frequency of spontaneous HR events compared to controls (Figure 2B). RAD51 has been shown to interact with BRCA2 at both the BRC repeat domain and c-terminal domain (Figure 1A) [4,8]. The constitutive and conditional removal of the BRC repeats in mice leads to early embryonic lethality, while incomplete removal allows for partial rescue, albeit with reduced Mendelian frequency and a propensity for tumor onset [5,6]. It is interesting to note that the fusion of a single BRC repeat (BRC3 or BRC4) to the single-stranded DNA-binding domain of RPA was able to restore *I-SceI* DSB-induced HR in the absence of BRCA2 [35]. Additionally, a purified fraction of BRCA2 containing all eight BRC repeats has been shown to stimulate RAD51 strand exchange in vitro [36]. Furthermore, a number of biochemical and structural studies suggest that BRC repeats of BRCA2 exon 11 bind to RAD51, affecting its cellular localization and function (e.g., RAD51 loading and filament formation) [37]. Lastly, Ayoub et al. used the DT-40 cell line to demonstrate that mutations in the c-terminal of BRCA2 that either abolished or enhanced RAD51 binding to BRCA2 did not affect HR frequency [38]. Therefore, these studies suggest an importance for some, if not all, BRC repeats of BRCA2 for proper HR.

The mouse model used in this study in which exon 11 is constitutively and/or conditionally removed should result in an in-frame deletion of BRC repeat containing exon 11. Though mRNA expression of the deletion allele has been observed [22], detection of the protein product has not been reported. If the BRCA2 exon 11 deletion protein is produced and is stable, then it should retain the RAD51 c-terminal interacting domain (Figure 1A). If this is in fact correct, then our results would suggest that the BRC repeats are necessary for promoting RAD51-dependent HR events and that the c-terminal RAD51 interacting domain is insufficient for this function of BRCA2. In support of this, we were able to detect RNA message and a protein band of approximately the correct predicted size in our *Brca2* delta 11 constitutive heterozygous animals (Figure 2B,C). However, it should be noted that the BRC repeats alone are probably not sufficient to permit BRCA2-dependent HR. Supporting this possibility is the observation by Edwards et al. that a secondary mutation in BRCA2, that restored HR activity, resulted in a truncated protein consisting of the first 5 BRC repeats and the c-terminus [39].

Finally, we wanted to determine whether conditional loss of *Brca2* exon 11 impacted HR events by reducing RAD51-dependent HR (e.g., gene conversions) while concomitantly increasing SSA events, as has been reported using cell line-based assays (e.g., mouse embryonic stem cells, V-C8 Chinese hamster ovary cells and CAPAN-1 pancreatic carcinoma cells) [11,12,40]. Based on previous studies from our laboratory, and from analogous studies in yeast, it is likely that the *p^un^* system detects both RAD51-dependent and –independent events (Figure 1A) [14,17,18]. We propose that spontaneous-induced (i.e., endogenous damage) multi-cell eye spots are the result of a reversion event that occurred in a proliferating cell, and as such, is most likely the product of RAD51-dependent HR (presumably at a stalled or collapse replication fork) (Figure 2A). Conversely, single-cell eye spots are not necessarily tied to DNA replication and are frequently the product of SSA HDR (Figure 2A). Gene conversion, but not SSA, has been shown to involve RAD51 [41] and loss of RAD51 both decreases gene conversions and increases SSA in mammalian tissue culture and yeast studies [12,42,43]. Previous tissue culture-based studies utilized recombination substrates to similarly compare different modes of HR repair when BRCA2 is absent or mutated. Those studies described that with reduced gene conversion in BRCA2-deficient cells, a concomitant increase in SSA was also observed [11,12,40]. We examined whether we observed this phenomenon too by quantifying the frequency of single- and multi-cell eye spots per RPE. As we would have predicted based on the prior tissue culture-based studies, we observed a decrease in multi-cell events (Figure 2E), suggesting a decrease in RAD51-dependent HR. In contrast to the tissue culture-based assays, we did not see any evidence of an increased frequency of SSA events as the frequency of single-cell events was also decreased compared to control (Figure 2D). This would suggest that the conditional loss of *Brca2* exon 11 did not lead to a hyper-SSA phenotype as measured by our in vivo system. A number of differences between our study and those mentioned could account for the discrepancy. The first difference being the type of BRCA2 mutation in which our system deleted all eight BRC repeats, potentially resulting in the expression of an isoform that retains the RAD51-interacting c-terminal domain (Figure 1). Secondly, the tissue culture-based HR assays were conducted in BRCA2 cell lines that also carried other genetic deficiencies whereas our mice are otherwise wild type. Of particular note is that in all other prior noted studies on the impact of BRCA2 mutation on HR the cell lines had compromised p53 function. V-C8 CHO cells were derived from V79 cells [44], and V79 cells are known to be p53 deficient [45]. In mouse ES cells, p53 protein appears to be cytoplasmically localized [46] and the p53-dependent responses appear compromised [47,48]. Similarly, CAPAN-1 cells are known to have a p53 mutation [49] in addition to their BRCA2 mutation. We believe that this point is significant since p53 nullizygosity was previously shown to result in an increased frequency of *p^un^* reversion [15,16] and recent work has highlighted the role of p53 in maintaining replication fork stability [50] via suppressing RAD52, a protein also known to promote SSA [51].

## 5. Conclusions

Overall, this study clearly demonstrates that BRCA2 is involved in promoting HR to repair spontaneous DNA damage that most likely resulted from stalled or collapsed replication forks. This is in addition to its established role in promoting HR to repair DSBs. In support of our findings, BRCA2 was found to have a role in the stabilization of stalled replication forks and preventing the accumulation of strand breaks that could potentially lead to malignant transformation [52]. With regard to the synthetic lethality of BRCAness tumors following PARP1 inhibition, this study, in light of our previous PARP1 deletion results [17], provides in vivo proof of principal as to how PARP1 inhibition is an effective therapeutic for BRCA2-deficient tumors that are impaired in HR as many cell-based, preclinical and clinical studies have demonstrated. Furthermore, our results show the importance of the BRC repeats for maintaining proper function of BRCA2 in HR, presumably through its interaction with RAD51. Going forward, this model could be used to investigate the in vivo consequences of PARP1 inhibition in the absence of BRCA2 either genetically or pharmacologically, as well as screen for compounds or genes that could restore HR in the absence of BRCA2, thereby providing insights into resistance mechanisms that need to be considered during treatment of these cancers.

## Figures and Tables

**Table 1:** Summary of RPEs examined and *p^un^* reversion frequency by genotype. Data represented are from the blue, >80% stain (Cre activity) samples. * The combined conditional (CC) control is the combination of the conditional control (*Trp1-Cre*) controls with *Brca2^wt/co11^*. The number of RPEs are the actual number of RPEs we examined, and the average number of eye spots per RPE is derived from the total number of eye spots divided by the number of RPEs examined.

Genotype	Number of RPE	Avg. # of Eye Spots per RPE
Conditional control	31	4.81
* CC control	41	4.76
*Brca2^wt/co11^*	10	4.6
*Brca2^∆11/co11^*	15	1.53
*Brca2^co/-^*	29	1.97

## Data Availability

Not applicable.

## Supplementary Materials

The following are available online at https://www.mdpi.com/article/10.3390/cancers13153663/s1, Figure S1: Original Western Blot Image.
