# Supplementary figures and images for: BRCA2 Promotes Spontaneous Homologous Recombination In Vivo
Source: Cancers (Basel). 2021 Jul 21;13(15):3663. doi: 10.3390/cancers13153663 (PMC8345144; doi:10.3390/cancers13153663)

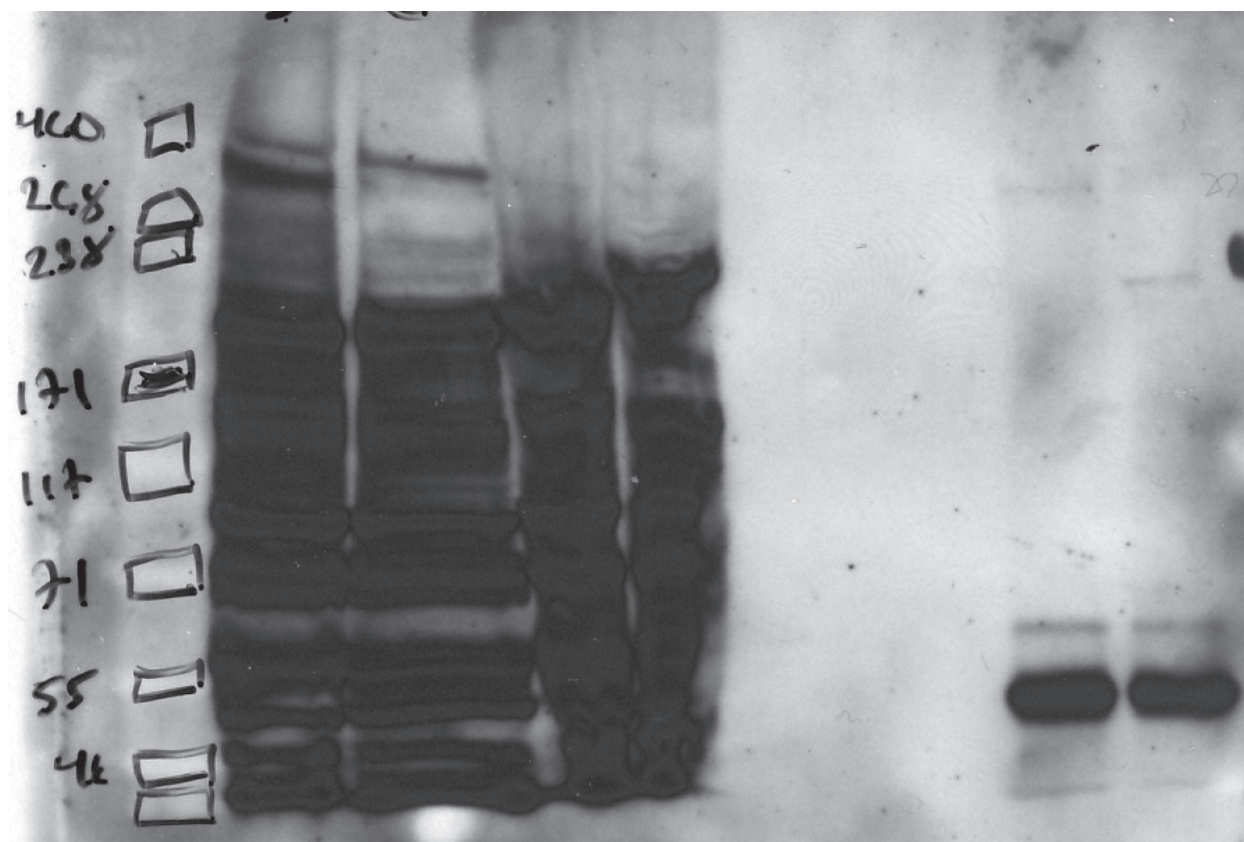

Figure S1. Original Western Blot Image.

Supplement: Supplementary file 1 [file cancers-13-03663-s001.zip › cancers-1258965-supplementary.pdf]
